# Preparation, Characterization and Evaluation of α-Tocopherol Succinate-Modified Dextran Micelles as Potential Drug Carriers

**DOI:** 10.3390/ma8105332

**Published:** 2015-09-28

**Authors:** Jingmou Yu, Yunfeng Zhou, Wencong Chen, Jin Ren, Lifang Zhang, Lu Lu, Gan Luo, Hao Huang

**Affiliations:** 1 School of Pharmacy and Life Sciences, Jiujiang University, 320 Xunyang East Road, Jiujiang 332000, China; zhou768417683@gmail.com (Y.Z.); chen393787420@gmail.com (W.C.); yanjiushengrj@gmail.com (J.R.); zhang524495632@gmail.com (L.Z.); yulu8275@gmail.com (L.L.); luo857084924@gmail.com (G.L.); 2 School of Chemical and Biological Engneering, Yichun University, 576 Xuefu Road, Yichun 336000, China

**Keywords:** dextran, α-tocopherol succinate, polymeric micelles, doxorubicin, drug carrier

## Abstract

In the present study, α-tocopherol succinate (TOS) conjugated dextran (Dex-TOS) was synthesized and characterized by fourier transform infrared (FT-IR) spectroscopy, ^1^H nuclear magnetic resonance (^1^H NMR), dynamic light scattering (DLS) and fluorescence spectroscopy. Dex-TOS could form nanoscaled micelles in aqueous medium. The critical micelle concentration (CMC) is 0.0034 mg/mL. Doxorubicin (Dox) was selected as a model drug. Dox-loaded Dex-TOS (Dex-TOS/Dox) micelles were prepared by a dialysis method. The size of Dex-TOS/Dox micelles increased from 295 to 325 nm with the Dox-loading content increasing from 4.21% to 8.12%. The Dex-TOS/Dox micelles were almost spherical in shape, as determined by transmission electron microscopy (TEM). *In vitro* release demonstrated that Dox release from the micelles was in a sustained manner for up to 96 h. The cellular uptake of Dex-TOS/Dox micelles in human nasopharyngeal epidermoid carcinoma (KB) cells is an endocytic process determined by confocal laser scanning microscopy (CLSM). Moreover, Dex-TOS/Dox micelles exhibited comparable cytotoxicity in contrast with doxorubicin hydrochloride. These results suggested that Dex-TOS micelles could be a promising carrier for drug delivery.

## 1. Introduction

Dextran, a polysaccharide consisting of 1, 6- and 1, 3-glucosidic linkages, has excellent water solubility and is widely used in medicine and biomedical devices [1,2,3]. Meanwhile, dextran and its derivatives have been extensively investigated for drug delivery vehicles due to biocompatibility, biodegradability and biological activities [4,5]. Hydrophobically modified dextrans have been successfully synthesized via derivatization of the primary hydroxyl groups. For example, Du and coworkers synthesized stearate-*g*-dextran (Dex-SA) amphiphiles [6]. Dox-loaded Dex-SA micelles could maintain the cytotoxicity of commercial doxorubicin injection against drug-sensitive tumor cells. Moreover, Dox-loaded Dex-SA micelles could effectively suppress the tumor growth and reduce the toxicity against animal body in nude mice bearing A549 human lung adenocarcinoma, compared with commercial doxorubicin injection. Hence, dextran-based polymeric amphiphiles have attracted significant attention as potential drug carriers.

Amphiphilic copolymer is consisting of hydrophilic and hydrophobic segments. It can form micelles or self-assembled nanoparticles via the intra- and/or intermolecular interactions of hydrophobic domain in aqueous medium [7,8,9,10]. Micelles exhibit unique characteristics, such as unusual rheological features, thermodynamic stability and structure of a hydrophobic core and a hydrophilic shell. Especially, they can enhance the solubility of hydrophobic drug, preferentially accumulate in tumor tissue via the enhanced permeation and retention (EPR) effect and reduce systemic side effects [11,12]. Among these nanocarriers, much attention has still been paid to prepare biodegradable and nontoxic polymeric micelles.

α-Tocopheryl succinate (TOS), one of the vitamin analogues, is a well-known hydrophobic molecule [13,14,15]. Furthermore, it has been approved as a pharmaceutical adjuvant or food supplement by the Food and Drug Administration and the European Medicine Agency [16]. Duhem *et al.* synthesized tocopherol succinate glycol chitosan (GC-TOS) conjugates. GC-TOS was non-cytotoxic at concentrations up to 10 mg/mL [17]. In addition, the result exhibited a 3.4-fold increase of the apparent permeation coefficient of ketoconazole across a Caco-2 cell monolayer. As reported by Mandracchia and co-workers, innovative inulin (INU)-vitamin E succinate (VITE) bioconjugates (INVITE) were synthesized and characterized [18]. Further, curcumin was selected as a model and curcumin-INVITE nanomicelles were prepared [19]. Pharmacokinetic studies on Balb/C mice exhibited the presence of curcumin up to 6 h in the blood, while “naked” curcumin is quickly removed from the bloodstream. More importantly, TOS can specifically destabilize cancer cell mitochondria but is nontoxic toward normal cells [20]. Considering the hydrophobicity and bioactive efficacy of TOS, we employed TOS chain to design functional copolymeric nanomaterial as the hydrophobic moieties. 

In this study, the objective is to construct novel α-tocopherol succinate conjugated dextran (Dex-TOS) nanocarriers. Dex-TOS conjugate was synthesized and characterized. Anticancer drug doxorubicin (Dox) was chosen as a model drug, which is widely used to treat different solid malignant tumors. Unfortunately, free Dox has severe side effects such as cardiotoxicity and myelosuppression in clinical application. The design and preparation of an excellent delivery system for effective deliver and release Dox into tumor cells is urgently needed. Therefore, Dox-loaded Dex-TOS micelles were prepared and characterized. Drug release from drug-loaded micelles was investigated. *In vitro* anti-tumor efficacy of Dox-loaded micelles was also studied in human nasopharyngeal epidermoid carcinoma (KB) cancer cells. 

## 2. Results and Discussion

### 2.1. Synthesis and Characterization of Dex-TOS Conjugate

**Figure 1:**
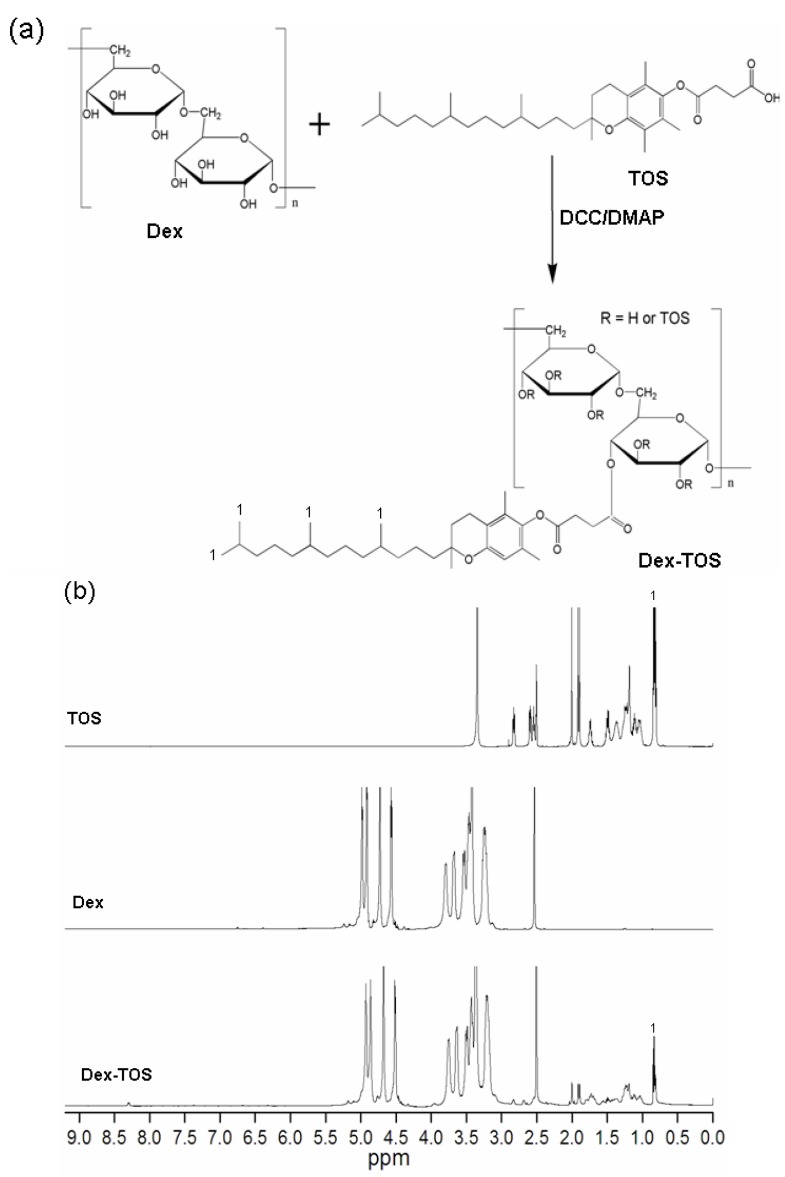
(**a**) Synthetic scheme of α-tocopherol succinate conjugated dextran (Dex-TOS) conjugate and (**b**) ^1^H nuclear magnetic resonance (^1^H NMR) spectra of α-tocopherol succinate (TOS), dextran (Dex) and Dex-TOS.

**Figure 2:**
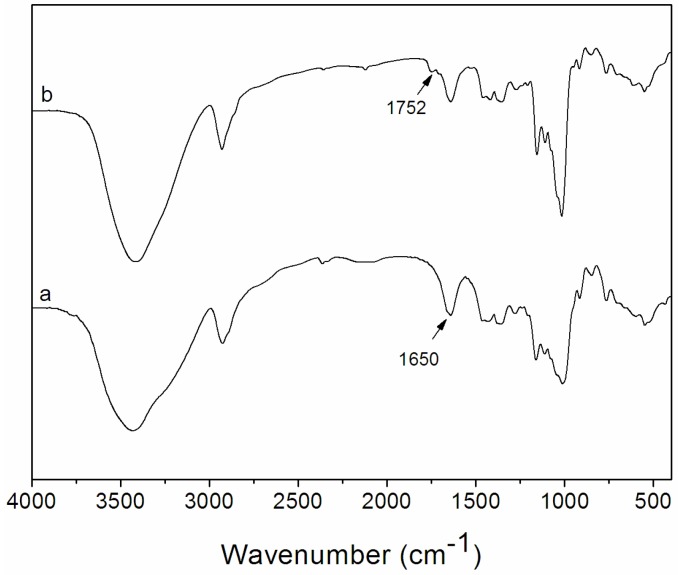
Fourier-transform infrared (FT-IR) spectra of (**a**) Dex and (**b**) Dex-TOS conjugate.

Dex-TOS conjugate was synthesized by the coupling reaction of carboxyl group of α-tocopherol succinate with hydroxyl group of dextran in the presence of *N*, *N*’-dicyclohexylcarbodiimide (DCC) and 4-dimethylaminopyridine (DMAP). The conjugation scheme of Dex-TOS is presented in Figure 1a. By this reaction, various Dex-TOS copolymers with different degree substitution (DS) of TOS were synthesized by controlling the feed ratio of TOS to Dex. The DS of Dex-TOS, determined by ultraviolet (UV) spectrophotometry, was 3.0% in this work. The DS of another Dex-TOS conjugate was 1.9%. Above this reaction feed ratio, the produced Dex-TOS conjugates (DS: >3.6%) could not form nanoscaled particles in aqueous media. The chemical structure of Dex-TOS is confirmed by analysis of FT-IR and ^1^H NMR spectra. Figure 2 shows the FT-IR spectra of dextran and Dex-TOS conjugate. For the Dex-TOS conjugation, as compared with dextran, new absorption band appeared at 1752 cm^−1^. The result was ascribed to the stretching of ester groups [21]. These results are evidence of the conjugation of TOS onto dextran.

The incorporation of TOS into dextran was further confirmed by ^1^H NMR. As shown in Figure 1b, specific peaks of dextran were observed at 3.0–3.8 ppm and 4.5–5.0 ppm [22]. Compared with Dex, new proton peaks of TOS groups in Dex-TOS copolymer appeared at 0.83–2.0, 2.60 and 2.81 ppm in ^1^H NMR spectrum (Figure 1b). These results further confirmed that Dex-TOS conjugates were successfully synthesized.

### 2.2. Determination of Critical Micelle Concentration (CMC)

**Figure 3:**
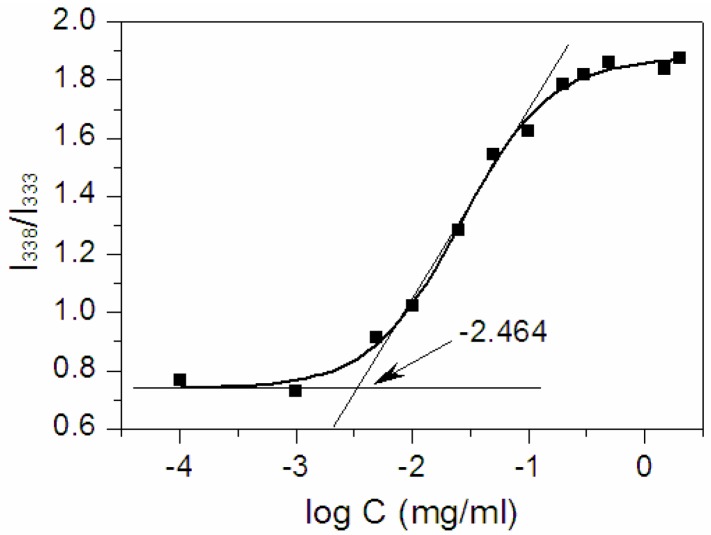
Polt of the intensity ratio *I*_338_/*I*_333_ (from pyrene excitation spectra of Dex-TOS) as a function of log C.

Amphiphilic copolymers are able to form micelles in aqueous media. The core–shell structure of micelles are made during the process. The CMC value is a parameter indicative of the micelle’s stability upon dilution. In order to determine the CMC of Dex-TOS, fluorescence measurement was carried out using pyrene as the hydrophobic fluorescence probe. Figure 3 showed the intensity ratio of I_338_/I_333_ *vs.* log C of Dex-TOS conjugate for the pyrene excitation spectra. A flat region at extremely low concentration and a sigmoid change in the crossover region were observed. CMC can be obtained from the intersection of two straight lines [23]. The CMC of Dex-TOS was calculated to be 0.0034 mg/mL, which was much lower than that of low molecular weight surfactants. The CMC value of another Dex-TOS conjugate (DS: 1.9%) was 0.0075 mg/mL. These results demonstrated that Dex-TOS conjugate was easy to form core–shell type nanoparticles in an aqueous environment. Moreover, we inferred that Dex-TOS micelles could remain stable even under highly diluted conditions and preserve their structure without dissociation *in vivo*.

### 2.3. Characterization of Blank and Dex-TOS/Dox Micelles

Blank micelles were prepared by dispersing Dex-TOS in an aqueous media. The particle size of Dex-TOS determined by dynamic light scattering (DLS) was 269 nm. Dox was encapsulated into Dex-TOS micelles by a dialysis method. The physicochemical properties of Dex-TOS/Dox micelles are summarized in Table 1. Based on the different ratios of feed drug to carrier (0.5/10, 1/10, 1.5/10, w/w), three kinds of Dex-TOS/Dox micelles were prepared, and named Dex-TOS-1/Dox, Dex-TOS-2/Dox and Dex-TOS-3/Dox, respectively. Their particle sizes determined by DLS were in the range of 295–325 nm. It was noticed that the hydrodynamic diameter was larger after loading the drug. This is due to the fact that the drug was solubilized inside the micelles. DOX was loaded into the micelles by the driving behavior of hydrophobic interaction, due to its low water solubility and interaction with the aromatic regions of TOS segments through π–π stacking [24]. This result was consistent with the report by Liang *et al.* [25]. Moreover, it was found that drug-loading content and particle size of Dex-TOS/Dox micelles increased as the weight ratio of Dox to Dex-TOS increased. The Dox-loading content increased from 4.21% to 8.12%, whereas the entrapment efficiency decreased from 87.9% to 58.9% at the same time. Based on the size and the drug loading property, the optimal ratio of drug to carrier (1.5/10) was selected to prepare Dox-loaded micelles. Therefore, Dex-TOS-3/Dox micelles (with a drug loading content of 8.12%) were used for the following studies.

**Table 1:** Physicochemical characteristics of Dex-TOS/Dox micelles.

Sample	Drug/Carrier ^a^	LC (%) ^b^	EE (%) ^c^	Size (nm) ^d^	PI ^e^
Dex-TOS-1/Dox	0.5/10	4.21	87.9	295 ± 12.3	0.223 ± 0.021
Dex-TOS-2/Dox	1/10	6.57	70.3	310 ± 21.4	0.126 ± 0.013
Dex-TOS-3/Dox	1.5/10	8.12	58.9	325 ± 25.6	0.128 ± 0.018

**^a^** The ratio of DOX to carrier, based on feed amount (mg/mg); **^b^** loading content; **^c^** encapsulation efficiency; **^d^** Measured by dynamic light scattering; **^e^** Polydispersity index.

**Figure 4:**
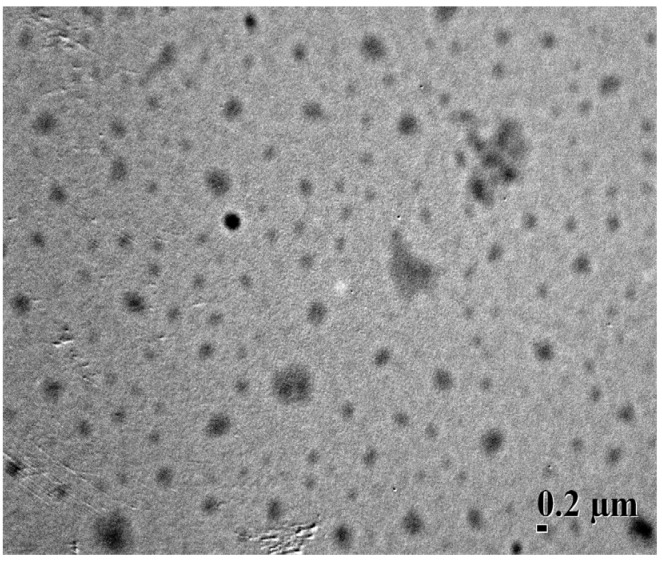
Transmission electron microscopy (TEM) image of Dex-TOS-3/Dox micelles (×10,000).

Transmission electron microscopy (TEM) image depicted that Dex-TOS-3/Dox micelles were spherical in shape (Figure 4). The size of Dex-TOS-3/Dox micelles determined by DLS was 325 nm, which was bigger than that observed by TEM. The difference was resulted from the sample preparation process. DLS analysis is in the hydrated state, while TEM sample was in the dried state. 

### 2.4. Differential Scanning Calorimetry (DSC)

**Figure 5:**
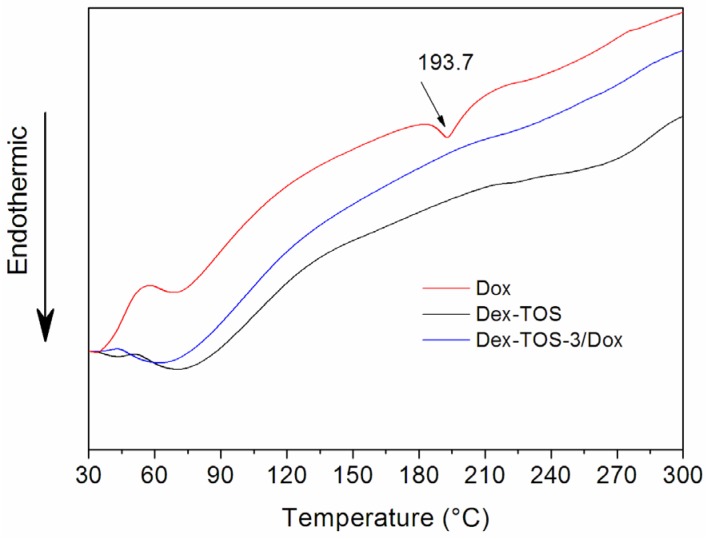
Differential scanning calorimetry (DSC) spectra of Dox, Dex-TOS, and Dex-TOS-3/Dox micelles.

To study the physical existing status of Dox in Dex-TOS/Dox micelles, DSC analysis was performed on Dox, blank Dex-TOS, and Dex-TOS-3/Dox micelles. In the DSC thermogram (Figure 5), the Dox powder and physical mixtures showed a melting endothermic peak at 193.7 °C. However, endothermic peak of Dox in the curve of Dex-TOS/Dox micelles had disappeared. These results implied that Dox was essentially amorphous or molecular state in the micelles [26].

### 2.5. In Vitro Release of Dox-Loaded Micelles

**Figure 6:**
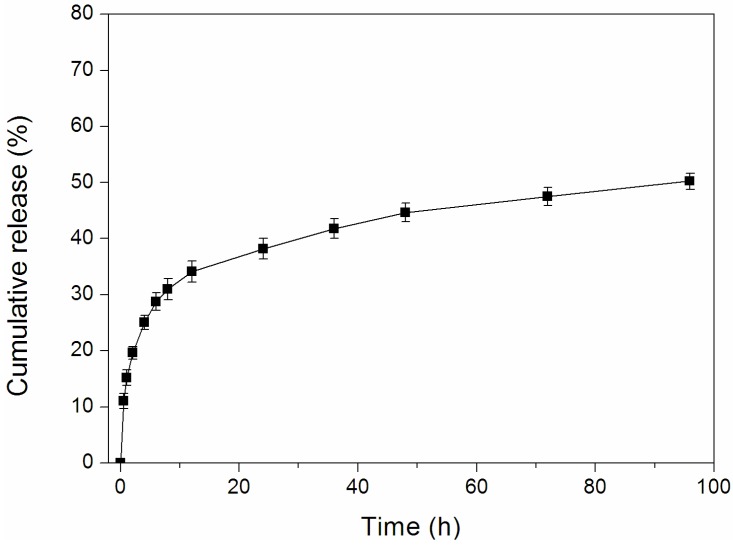
Release profile of Dox from Dex-TOS-3/Dox micelles at 37 °C in phosphate buffer solution (PBS) at pH 7.4.

The release of Dox from Dex-TOS/Dox micelles was performed in phosphate buffer solution (PBS) at pH 7.4, simulating the *in vivo* biological environment. The cumulative release profiles of Dox from the micelles were plotted in Figure 6. Dex-TOS-3/Dox micelles displayed the initial burst release for about 12 h and subsequent sustained release. The total amount of drug released from Dex-TOS-3/Dox was 50.2% over 96 h. The slow drug release was affected by either hydrophobic–hydrophobic interactions or van der Waals interactions between Dox molecules and the hydrophobic groups of the conjugates.

### 2.6. Confocal Laser Scanning Microscopy (CLSM) Observation 

**Figure 7:**
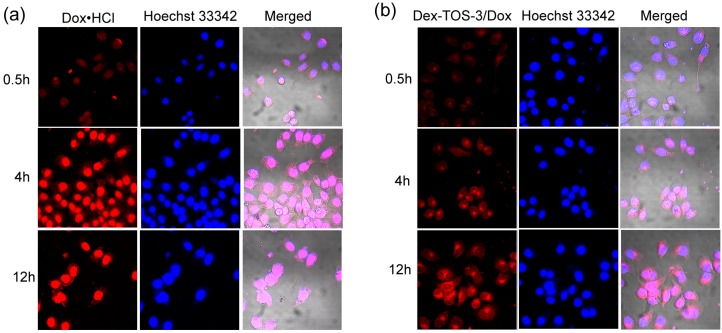
Confocal laser scanning microscopy (CLSM) images of human nasopharyngeal epidermoid carcinoma (KB) cells after incubation with (**a**) doxorubicin hydrochloride (Dox·HCl) and (**b**) Dex-TOS-3/Dox micelles for 0.5, 4 and 12 h.

The intracellular fate of Doxorubicin hydrochloride (Dox·HCl) or Dex-TOS-3/Dox micelles was monitored in KB cells by CLSM. As shown in Figure 7, the red fluorescence was emitted from Dox formulations and the blue fluorescence stained in nuclei was from Hoechst 33342. Dox·HCl incubation for 0.5 h showed red fluorescence mainly in the nuclei, while Dox fluorescence from Dex-TOS-3/Dox micelles was distributed mostly in cytoplasm. By contrast, red fluorescence emitted from Dox·HCl was stronger than that of Dex-TOS-3/Dox micelles. With further incubation for 4 h, Dox fluorescence intensities of these Dox formulations increased inside the cells. The red fluorescence from Dox·HCl in the nuclei became more evident. In addition, the cells incubated with Dex-TOS-3/Dox micelles presented strong Dox fluorescence in the cytoplasm, and some fluorescence dots observed in the nuclei. After 12 h incubation, red intensities from both Dox formulations were higher than that for 4 h. In addition, Dox·HCl exhibited higher fluorescence than Dex-TOS-3/Dox micelles. Notably, it can be found that the fluorescence from Dex-TOS-3/Dox micelles significantly increased in the nuclei. These different results were ascribed to the different internalization ways between Dox·HCl and Dex-TOS-3/Dox micelles. Dox·HCl was entered into the cells in a passive diffusion way, whereas Dox-loaded micelles were transported into the cells by endocytosis manner [27]. Moreover, Dex-TOS-3/Dox micelles exhibited large particle size and high molecular weight. Dox-loaded micelles were difficult to directly transport into the nuclei [28,29]. The red fluorescence from Dex-TOS-3/Dox micelles in nuclei was mostly attributed to the release of free Dox molecules. As described above, Dox release from drug-loaded micelles was in a sustained way. Therefore, the Dox accumulation in the nucleus from Dox·HCl was higher than that from Dox-loaded micelles in 12 h. Interestingly, TOS has the mitochondrial targetability and can participate in the mitochondrial signaling pathways. Thus high Dox fluorescence of Dex-TOS-3/Dox micelles distributed in the cytoplasm was beneficial to the improvement of apoptotic cell death. In addition, the experiments were performed in 24 h incubation (images not shown). However, great amount of cancer cells were dead in the plates of Dox·HCl and Dex-TOS-3/Dox micelles.

### 2.7. In Vitro Cytotoxicity Study

**Figure 8:**
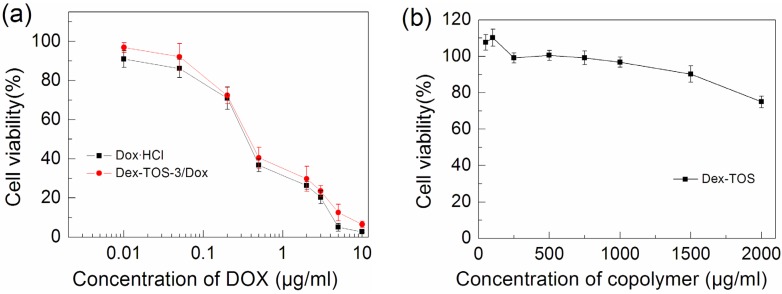
The cytotoxicity of (**a**) Dox·HCl, Dex-TOS-3/Dox micelles, and (**b**) blank Dex-TOS micelles against KB cells after 48 h incubation.

The *in vitro* cytotoxicity of both Dox-loaded micelles and Dox·HCl against KB cells were determined using 3-(4, 5-dimethyl-thiazol-2-yl)-2, 5-diphenyl-tetrazolium bromide (MTT) assay. As shown in Figure 8a, Dox formulations showed dose-dependent cytotoxicity to KB cells. Dox·HCl and Dex-TOS-3/Dox micelles exhibited comparable cytotoxicity to KB cells at the same dose of Dox. The IC_50_ of Dox·HCl and Dex-TOS-3/Dox micelles was 0.31 and 0.35 μg/mL, respectively. As previously reported [30], Dox·HCl can be quickly transported into cells by passive diffusion, while Dox-loaded micelles can be internalized in cells by endocytosis and Dox release is time-consuming process. As described above, only 45% of Dox were released from the drug carrier in 48 h. The released Dox contributed to the cytotoxicity of Dox-loaded micelles. The actual cytotoxicity of Dox-loaded micelles should be greater along with the extension of time [31]. Notably, TOS can target mitochondria, leading to promote the mitochondria-specific apoptotic pathways [20]. Therefore, Dex-TOS can synergistically maximize the cytotoxic efficacy of the loaded DOX in the micelles via the targeting mitochondria. It was known that drug-loaded micelles can prevent premature drug release in blood and efficiently deliver it to tumor cells by EPR effect. Hence, it was speculated that the higher concentration of Dox from Dox-loaded micelles would be present in tumor site or tumor cells than that from Dox·HCl after intravenous injection of the same dose of Dox. In addition, the cell viabilities of blank Dex-TOS micelles against KB cells were ≥95%, up to a concentration of 1 mg/mL (Figure 8b). These results revealed that Dex-TOS micelles were practically nontoxic and had good biocompatibility.

## 3. Experimental Section

### 3.1. Materials

Dextran (Dex, 40 kDa), α-tocopherol succinate (TOS), *N*, *N*’-dicyclohexylcarbodiimide (DCC), and 4-dimethylaminopyridine (DMAP) were purchased from Sigma-Aldrich (St. Louis, MO, USA). Anhydrous dimethyl sulfoxide (DMSO) and pyrene were provided by Acros Organics (Beijing, China). Doxorubicin hydrochloride (Dox·HCl) was obtained from Beijing Huafeng United Technology Co., Ltd. (Beijing, China). Human nasopharyngeal epidermoid carcinoma (KB) cells were purchased from the Institute of Biochemistry and Cell Biology of Chinese Academy of Sciences (Shanghai, China). RPMI 1640 medium and trypsin-EDTA were obtained from Jinuo biotechnology company (Hangzhou, China). Fetal bovine serum (FBS) was from Sijiqing Biologic Co. Ltd. (Zhejiang, China). 3-(4, 5-Dimethyl-thiazol-2-yl)-2, 5-diphenyl-tetrazolium bromide (MTT) was purchased from Sigma-Aldrich (St. Louis, MO, USA). All other chemicals were of analytical grade.

### 3.2. Synthesis of Dex-TOS Conjugate

Five hundred milligrams dextran and 60 mg DMAP were dissolved in 15 mL anhydrous DMSO. TOS (327 mg) and DCC (382 mg) were dissolved in 100 mL anhydrous DMSO and stirred for 4 h under the protection of nitrogen. TOS/DCC solution was slowly added to the dextran/DMAP solution. This mixed solution was stirred to react for 48 h in nitrogen gas at room temperature. After that, this solution was filtered to remove the byproducts and dialyzed against distilled water for 2 days. The resultant solution was freeze-dried. Dextran is insoluble in anhydrous ethanol. The lyophilized product was dispersed in anhydrous ethanol under the help of sonication to remove unreacted TOS. Then the mixture was centrifuged at 12,000 rpm for 5 min (Sigma laboratory centrifuges 3K18, Osterode, Germany). The ethanol solution was discarded. The precipitated product was dissolved in water and dialyzed against distilled water for another 2 days. The dialyzed solution was filtered through a 0.8-μm membrane and lyophilized. The white product Dex-TOS was obtained.

FT-IR spectrum of Dex-TOS was recorded on a Fourier-transform infrared spectrometer (Vertex 70, Bruker Corporation, Ettlingen, Germany). Dex or Dex-TOS was thoroughly ground with exhaustively dried KBr. The composition of Dex-TOS was further confirmed by ^1^H NMR spectra using a NMR spectrometer (Avance DMX500, Bruker Corporation, Ettlingen, Germany). DMSO-*d*6 was used as solvent.

The substitution degree (DS) of TOS to dextran was expressed as mol % *vs.* 100 glucose groups. The extent of TOS conjugation was determined by ultraviolet (UV) spectrophotometry as previously described by Mandracchia *et al.* [18]. The absorbance wavelength is set at 286 nm. The calibration curve of TOS in DMSO was made in the concentration range 60–240 μg/mL (*R*^2^ = 0.9962). Twenty-seven milligrams of Dex-TOS were dissolved in 15 mL DMSO. The absorbance value was analyzed by an UV spectrophotometer (TU-1901, Beijing Purkinje General Instrument Co., Ltd., Beijing, China).

### 3.3. Determination of Critical Micelle Concentration (CMC)

Fluorescence spectroscopy was employed to study the micellization property of Dex-TOS. Briefly, Dex-TOS conjugate suspension was adjusted to various concentrations. A known amount of pyrene in acetone was added to 10-mL vials, and acetone was evaporated at 40 °C. Then, 10 mL of various concentrations of sample suspension were added to each vial, and heated at 50 °C for 10 h to equilibrate the pyrene and the micelles, and remained undisturbed to cool overnight at room temperature. The final concentration of pyrene was 6.0 × 10^−7^ M. Steady-state fluorescent spectra were measured by fluorescence spectrophotometer (Hitachi F-4500, Hitachi Ltd., Tokyo, Japan) with a slit width of 2.5 nm. The excitation and emission wavelength was set at 339 and 390 nm, respectively.

### 3.4. Preparation of Dox-loaded Micelles

The Dox-loaded Dex-TOS micelles were prepared by a dialysis method. Briefly, Dex-TOS conjugate (100 mg) was dissolved in 2 mL of DMSO. Different amounts of doxorubicin hydrochloride (5, 10 or 15 mg) were separately dissolved in 2 mL DMSO with 3 equivalent molar ratio of triethylamine, and stirred overnight under the dark condition. Then Dox solution was added to the above Dex-TOS solution. The mixture solution was magnetically stirred for 6 h, and placed into the dialysis bag (MWCO: 7 kDa) for dialysis against distilled water for 24 h. After that, the dialysis solution was filtered through a 0.8 μm membrane and lyophilized.

### 3.5. Characterization of Blank and Dex-TOS/Dox Micelles

The mean diameter and size distribution of blank and Dex-TOS/Dox micelles were analyzed by dynamic light scattering (90Plus, Brookhaven Instruments Corp., New York, NY, USA). The morphology of Dex-TOS micelles encapsulating Dox was observed using a transmission electron microscopy (JEM-1230, Jeol, Tokyo, Japan). A drop of sample solution was placed onto a 300-mesh copper grid coated with carbon and the extra solution was blotted with filter paper, followed by air-drying. Observation was performed at 80 kV.

The drug loading content (*LC*) and encapsulation efficiency (*EE*) of Dex-TOS/Dox micelles were determined by extracting the drug from the micelles and using UV method. Dex-TOS/Dox micelles were dissolved in aqueous solution and disrupted by the addition of DMSO. Dox concentration was measured using an UV spectrophotometer at 481 nm. The *LC* and *EE* of Dex-TOS/Dox micelles were calculated with the following equations:

(1) $$LC\;(\%)\;=\frac{\text{Dox}\;\text{weight}\;\text{in}\;\text{the}\;\text{micelles}}{\text{weight}\;\text{of}\;\;\text{Dex}-\text{TOS}/\text{Dox}\;\text{micelles}}\times 100$$

(2) $$EE\;(\%)\;=\frac{\text{Dox}\;\text{weight}\;\text{in}\;\text{the}\;\text{micelles}}{\text{feed}\;\text{Dox}\;\text{weight}}\times 100$$

### 3.6. DSC Analysis

In order to evaluate the status of Dox in Dex-TOS/Dox micelles, DSC analysis was performed by STA 8000 Thermal Analyzer (PerkinElmer, Waltham, MA, USA). Dox, Dex-TOS or Dex-TOS/Dox micelles were analyzed at heating temperature ranging from 30 to 300 °C with a rate of 10 °C min. 

### 3.7. In Vitro Release of Dox-loaded Micelles

Dox release from Dox-loaded micelles was investigated *in vitro* by a dialysis method in phosphate buffered saline (PBS, pH 7.4). Briefly, 1 mL of Dox-loaded micelles was placed in a dialysis bag (MWCO: 7 kDa) and dialyzed against 20 mL of PBS medium. The condition was set at 37 °C and maintained at 100 rpm in an air-bath shaker. At predetermined time intervals, the total sample was removed and replaced with 20 mL of fresh PBS media. The amount of Dox released from the micelles was investigated by fluorescence spectrophotometer (Hitachi F-4500, Hitachi Ltd., Tokyo, Japan). An excitation wavelength of 470 nm and an emission wavelength of 585 nm were applied.

### 3.8. Confocal Laser Scanning Microscopy (CLSM) Observation

CLSM was adopted to observe the intracellular distribution of Dox. Briefly, KB cells were seeded at a density of 3 × 10^5^ cells/well in 6-well plates (Costar, Corning, NY, USA) overnight at 37 °C in 5% CO_2_. After removed the cultured media, Dox·HCl or Dex-TOS/Dox micelles (final Dox concentration: 10 μg/mL) in RPMI 1640 medium supplemented with 10% FBS were added. After 0.5, 4 or 12 h incubation, the cells were washed three times with PBS (pH 7.4) and fixed in 4% paraformaldehyde solution for 10 min. Further, the cells were treated with Hoechst 33342 (5 μg/mL) for nuclei staining. After 20 min, KB cells were washed with PBS. The cells were examined by a Zeiss LSM-510 confocal microscope (ZEISS, Jena, Germany) under the identical settings.

### 3.9. In Vitro Cytotoxicity Study

The cytotoxicity of blank or Dox-loaded micelles was evaluated *in vitro* by the MTT assays. KB cells were seeded at a density of 4 × 10^3^ cells/well in 96-well plates and cultured overnight. Then, the culture medium was removed. Various concentrations of blank micelles, Dox·HCl, or Dex-TOS/Dox micelles which were dispersed in RPMI 1640 medium containing 10% FBS were added. After 48 h treatment, the medium in each well was discarded. One hundred milliliters of fresh culture medium and 20 μL of MTT solution (5 mg/mL in PBS) were added. The cells were incubated for 4 h, and the medium was replaced by 150 μL DMSO to dissolve the purple crystals. After 15 min, the optical densities at 570 nm were measured with a microplate reader (Thermo Fisher Scientific, Waltham, MA, USA). Cells cultured in RPMI 1640 medium containing 10% FBS were used as controls.

## 4. Conclusions

Dex-TOS conjugate was successfully synthesized and characterized. The Dex-TOS conjugate could self-assemble to form nanosized micelles in aqueous medium and show low CMC. The release profile of Dex-TOS/Dox micelles showed that Dox could be released rapidly in the first 12 h and the sustaining release could last for about 96 h. Dox-loaded micelles could be internalized in KB cells by endocytosis manner. Dex-TOS/Dox micelles exhibited comparable cytotoxicity against KB cells in comparison with Dox·HCl. Furthermore, blank Dex-TOS micelles showed good biocompatibility. Taken together, the results suggest that Dex-TOS micelles have potential as drug delivery vehicles for cancer therapy.
