# Correction: Preparation, Characterization and Evaluation of α-Tocopherol Succinate-Modified Dextran Micelles as Potential Drug Carriers. *Materials* 2015, *8*, 6685–6696

**DOI:** 10.3390/ma8125448

**Published:** 2015-12-01

**Authors:** Jingmou Yu, Yunfeng Zhou, Wencong Chen, Jin Ren, Lifang Zhang, Lu Lu, Gan Luo, Hao Huang

**Affiliations:** 1 School of Pharmacy and Life Sciences, Jiujiang University, 320 Xunyang East Road, Jiujiang 332000, China; zhou768417683@gmail.com (Y.Z.); chen393787420@gmail.com (W.C.); yanjiushengrj@gmail.com (J.R.); zhang524495632@gmail.com (L.Z.); yulu8275@gmail.com (L.L.); luo857084924@gmail.com (G.L.); 2 School of Chemical and Biological Engneering, Yichun University, 576 Xuefu Road, Yichun 336000, China

The authors wish to make the following correction to their paper [1]:

The second author’s name “Yufeng Zhou” should be “Yunfeng Zhou”.

The authors would like to apologize for any inconvenience caused to the readers by this error.
